# Supplementary material for: Production of alkanes from CO2 by engineered bacteria
Source: Biotechnol Biofuels. 2018 Aug 21;11:228. doi: 10.1186/s13068-018-1229-2 (PMC6102805; doi:10.1186/s13068-018-1229-2)
Supplement: Supplementary file 1 — Additional file 1. Nucleotide sequences. [file 13068_2018_1229_MOESM1_ESM.docx]

Additional File 1: Nucleotide sequences

**Production of alkanes from CO2 by engineered bacteria**

Tapio Lehtinen^1^*, Henri Virtanen^1^, Suvi Santala^1^, Ville Santala^1^

^1^ Tampere University of Technology, Department of Chemistry and Bioengineering, Korkeakoulunkatu 8, FI-33720, Tampere, Finland

* Corresponding author, [tapio.lehtinen@tut.fi](mailto:tapio.lehtinen@tut.fi)

The nucleotide sequences of the alkane biosynthesis genes utilized in the study are provided below.

***seado*** (*S. elongatus* *ado*, WP_011378104.1)

ATGCCACAACTGGAAGCGTCGTTGGAACTGGATTTTCAATCGGAAAGCTATAAAGATGCGTATTCACGTATCAATGCCATCGTCATTGAAGGTGAACAGGAAGCATTTGATAACTATAACCGTTTGGCAGAAATGCTGCCGGATCAGCGCGATGAACTGCATAAATTAGCGAAAATGGAACAACGTCACATGAAAGGTTTTATGGCCTGTGGCAAAAATTTGAGCGTAACCCCAGATATGGGTTTTGCGCAGAAATTTTTCGAACGCTTGCATGAAAACTTTAAAGCAGCAGCAGCAGAAGGCAAAGTGGTTACATGTCTGTTGATCCAAAGTCTGATCATCGAATGCTTTGCAATCGCAGCGTATAATATTTATATCCCTGTTGCCGATGCTTTTGCACGTAAAATTACTGAAGGTGTAGTCCGTGATGAATATCTGCATCGCAATTTTGGCGAAGAATGGTTAAAAGCTAACTTTGATGCGAGTAAAGCCGAACTGGAAGAAGCCAATCGTCAAAACTTGCCGCTGGTGTGGTTAATGTTGAATGAAGTTGCTGATGATGCACGTGAATTAGGCATGGAACGCGAATCTTTGGTGGAAGATTTTATGATTGCGTATGGCGAAGCCCTGGAAAACATTGGCTTTACAACCCGTGAAATCATGCGTATGAGTGCGTATGGTCTGGCAGCAGTTTAA

***pmado*** (*P. marinus* *ado*, WP_011130600.1)

ATGCCTACCTTAGAAATGCCGGTAGCAGCGGTCTTGGATTCAACTGTAGGTAGTTCTGAAGCACTGCCGGATTTTACGAGCGATCGTTATAAAGATGCTTATTCACGCATTAATGCGATTGTGATCGAAGGTGAACAGGAAGCGCATGATAACTATATTGCCATCGGCACCCTGTTACCAGATCATGTCGAAGAATTAAAACGTTTGGCGAAAATGGAAATGCGCCATAAGAAAGGTTTTACAGCCTGTGGTAAAAATCTGGGCGTGGAAGCAGATATGGATTTTGCACGTGAATTTTTCGCCCCACTGCGCGATAACTTTCAGACTGCGTTAGGTCAAGGCAAAACCCCTACATGCTTGCTGATTCAGGCACTGTTGATCGAAGCATTTGCGATTTCGGCATATCATACGTATATCCCTGTTAGTGATCCATTTGCCCGTAAAATTACCGAAGGTGTGGTTAAAGATGAATATACACATCTGAACTATGGCGAAGCCTGGCTGAAAGCTAACTTAGAATCGTGTCGTGAAGAACTGTTAGAAGCTAATCGCGAAAACTTGCCTCTGATTCGTCGCATGTTGGATCAGGTGGCAGGTGATGCCGCTGTTTTACAAATGGATAAAGAAGATTTGATCGAAGATTTTCTGATTGCGTATCAGGAATCTCTGACAGAAATCGGCTTTAATACTCGTGAAATTACGCGCATGGCAGCAGCAGCACTGGTGAGCTAA

***cer1*** (*Arabidopsis thaliana*, NP_001184890.1)

ATGGCAACCAAACCTGGCGTGTTAACTGATTGGCCTTGGACGCCGTTGGGTTCATTTAAATATATTGTGATCGCTCCGTGGGCAGTTCATTCGACCTATCGTTTTGTTACAGATGATCCTGAAAAACGCGATTTAGGTTATTTTCTGGTATTTCCGTTTCTGTTGTTTCGTATTCTGCATAATCAGGTCTGGATCTCTTTAAGCCGCTATTATACCAGTTCTGGCAAACGTCGCATTGTGGATAAAGGTATCGATTTTAACCAGGTTGATCGTGAAACAAATTGGGATGATCAAATCCTGTTTAACGGTGTGCTGTTTTATATTGGCATCAATTTGCTGCCAGAAGCTAAACAACTGCCTTGGTGGCGTACCGATGGCGTGCTGATGGCAGCGTTAATTCATACAGGTCCTGTTGAATTTTTGTATTATTGGCTGCATAAAGCATTACATCATCATTTTCTGTATTCTCGTTATCATAGCCATCATCATAGTAGTATTGTGACCGAACCGATTACAAGCGTCATCCATCCATTTGCGGAACATATTGCCTATTTTATCCTGTTTGCAATCCCATTGTTGACCACACTGTTAACTAAAACGGCGAGTATTATCTCTTTTGCCGGCTATATCATCTATATCGATTTTATGAACAACATGGGTCATTGTAACTTTGAACTGATCCCGAAACGTTTATTTCATTTGTTTCCGCCACTGAAATTTTTGTGCTATACTCCAAGCTATCATTCACTGCATCATACTCAGTTTCGCACGAATTATAGTTTGTTTATGCCGCTGTATGATTATATCTATGGCACTATGGATGAATCTACCGATACATTGTATGAAAAAACGCTGGAACGTGGTGATGATATCGTGGATGTGGTTCATTTGACACATCTGACTACGCCAGAAAGTATTTATCATTTACGCATCGGCTTGGCGTCGTTTGCCAGTTATCCATTTGCGTATCGTTGGTTTATGCGCTTGCTGTGGCCTTTTACTTCACTGTCGATGATTTTTACGCTGTTTTATGCGCGTTTGTTTGTGGCCGAACGCAATTCGTTTAACAAACTGAATTTACAGAGTTGGGTTATCCCGCGTTATAATCTGCAATATTTATTGAAATGGCGCAAAGAAGCAATCAACAACATGATCGAAAAAGCTATCTTAGAAGCAGATAAGAAAGGTGTGAAAGTCTTGAGCCTGGGTTTAATGAACCAAGGCGAAGAACTGAATCGTAACGGCGAAGTTTATATCCATAACCATCCAGATATGAAAGTACGTCTGGTCGATGGTAGTCGTCTGGCAGCAGCAGTGGTCATTAACAGCGTACCTAAAGCTACCACATCAGTGGTTATGACCGGCAATCTGACAAAAGTCGCATATACCATTGCGTCTGCCTTATGTCAGCGTGGTGTGCAAGTTAGCACATTGCGCCTGGATGAATATGAAAAAATTCGTAGCTGTGTACCGCAGGAATGCCGCGATCATTTAGTCTATTTGACCTCAGAAGCGCTGTCGAGTAATAAAGGCTTTTGGGTAAAAGTCTGGTTAGTGGGTGAAGGCACTACGCGTGAAGAACAAGAAAAAGCCACTAAGGGTACGCTGTTTATTCCGTTTTCTCAGTTTCCACTGAAACAATTACGTCGCGATTGTATTTATCATACCACACCTGCGTTGATCGTACCGAAAAGTCTGGTGAACGTTCATTCTTGCGAAAATTGGCTGCCACGTAAAGCTATGAGTGCAACTCGCGTTGCTGGTATTTTGCATGCACTGGAAGGCTGGGAAATGCATGAATGTGGTACGTCACTGTTATTGTCGGATCTGGATCAGGTTTGGGAAGCCTGCTTATCTCATGGTTTTCAACCACTGTTATTGCCTCATCATTAA

***aar*** (*S. elongatus aar,* WP_011242364.1)

ATGTTTGGTTTAATTGGCCATTTGACCTCGCTGGAACAGGCACGTGATGTTAGTCGTCGCATGGGCTATGATGAATATGCGGATCAGGGTCTGGAATTTTGGAGTTCTGCCCCGCCACAAATTGTGGATGAAATCACCGTTACATCTGCTACAGGCAAAGTGATTCATGGTCGTTATATCGAAAGCTGTTTTCTGCCGGAAATGCTGGCAGCGCGTCGCTTTAAAACTGCCACGCGCAAAGTTTTAAATGCTATGAGTCATGCACAGAAACATGGTATTGATATCAGCGCGCTGGGTGGCTTTACCTCAATCATCTTTGAAAACTTTGATTTAGCCTCTTTGCGTCAAGTACGCGATACCACACTGGAATTTGAACGTTTTACTACGGGCAACACCCATACAGCGTATGTAATTTGTCGCCAGGTCGAAGCCGCTGCAAAAACTCTGGGTATTGATATCACTCAAGCGACGGTTGCCGTGGTTGGTGCTACGGGCGATATTGGTTCGGCAGTATGCCGTTGGTTGGATCTGAAATTAGGTGTCGGCGATTTGATTCTGACTGCTCGTAATCAGGAACGCCTGGATAACTTACAAGCAGAACTGGGTCGTGGCAAAATTTTACCTTTGGAAGCGGCCTTACCGGAAGCGGATTTTATTGTATGGGTCGCCTCAATGCCGCAGGGTGTAGTCATCGATCCAGCAACATTAAAACAACCTTGCGTGTTGATTGATGGTGGCTATCCAAAAAATTTGGGTAGCAAAGTGCAAGGTGAAGGCATTTATGTTCTGAACGGTGGCGTGGTTGAACATTGTTTTGATATTGATTGGCAGATCATGAGTGCAGCAGAAATGGCACGTCCTGAACGCCAAATGTTTGCATGCTTTGCGGAAGCCATGCTGTTAGAATTTGAAGGCTGGCATACCAATTTTTCATGGGGTCGTAACCAGATTACAATCGAAAAAATGGAAGCGATCGGCGAAGCCTCGGTGCGTCATGGTTTTCAACCGCTGGCTTTAGCAATTTAA

***acr1*** *(A. baylyi ACIAD3383)*

ATGATATCAATCAGGGAAAAACGCGTGAACAAAAAACTTGAAGCTCTCTTCCGAGAGAATGTAAAAGGTAAAGTGGCTTTGATCACTGGTGCATCTAGTGGAATCGGTTTGACGATTGCAAAAAGAATTGCTGCGGCAGGTGCTCATGTATTATTGGTTGCCCGAACCCAAGAAACACTGGAAGAAGTGAAAGCTGCAATTGAACAGCAAGGGGGACAGGCCTCTATTTTTCCTTGTGACCTGACTGACATGAATGCGATTGACCAGTTATCACAACAAATTATGGCCAGTGTCGATCATGTCGATTTCCTGATCAATAATGCAGGGCGTTCGATTCGCCGTGCCGTACACGAGTCGTTTGATCGCTTCCATGATTTTGAACGCACCATGCAGCTGAATTACTTTGGTGCGGTACGTTTAGTGTTAAATTTACTGCCACATATGATTAAGCGTAAAAATGGCCAGATCATCAATATCAGCTCTATTGGTGTATTGGCCAATGCGACCCGTTTTTCTGCTTATGTCGCGTCTAAAGCTGCGCTGGATGCCTTCAGTCGCTGTCTTTCAGCCGAGGTACTCAAGCATAAAATCTCAATTACCTCGATTTATATGCCATTGGTGCGTACCCCAATGATCGCACCCACCAAAATTTATAAATACGTGCCCACGCTTTCCCCAGAAGAAGCCGCAGATCTCATTGTCTACGCCATTGTGAAACGTCCAAAACGTATTGCGACGCACTTGGGTCGTCTGGCGTCAATTACCTATGCCATCGCACCAGACATCAATAATATTCTGATGTCGATTGGATTTAACCTATTCCCAAGCTCAACGGCTGCACTGGGTGAACAGGAAAAATTGAATCTGCTACAACGTGCCTATGCCCGCTTGTTCCCAGGCGAACACTGGTAA

***ramo*** *(Nevskia ramosa* WP_022976613.1)

ATGAATTACTTCGTCACCGGCGCCACCGGTTTCATCGGCAAGCATCTGATCGAGCGCCTGCTGGCGCGCCCGGATGCCACCATCCACGTGCTGGTCCGCGCATCTTCGGAAGACAAGTTCGCCGCCTTGCAGGAGCGCTACGGTGATGCCGGCGACCGGCTGCAGATGGTCGCTGGCGACATCACCACGCCGGGCCTGGTGTCCGCCGCGGAGCTCAAGAAGCTGAAGGGCAAGGTCGGACACGTGTTTCACCTGGCCGCCGTCTACGACATGAACATGGACGATGCGACCGGCGATCGCATCAACAACGAAGGCACGCGCAACACCGTGGCCTTCGCCAACAGTCTGGGCGGCGACGTGGTGCTGCATCACGTGTCGAGCGTGGCGGTGGCCGGCGGCGATTTCGTCGGCACTTTCACCGAAGCGATGTTCGACGAAGGCCAGCCGGTCAAGCATCCGTACTTCCGGACCAAGTTCCAGTCCGAGAAGATCGTCCGTGACGAAGCCAAGGTGCCGTTCCGGGTCTATCGTCCCGGTGCTGTTGTCGGCCATTCGAAGACCGGCGAGATGGACAAGATCGACGGCCCTTACTACTTCTTCAAGACCATCCAGAAGCTCAGCCACCGGATTCCGAAGTGGCTGCCACTGCTCGGCATCGAAGGCGGCAAGGTGCCGATCGCGCCGGTCGACTACATCGCCGATGCGCTCGATGCGATCGCCCACAAGGACGGCCTGAACGGCCAGACCTTCCATCTGGTGCAGTCGAACAGCCCCAGCGTCGGCGACCTGATCCAGTCGATCCTGAAGGCCGCCCACGGGCCGCGCTTCAAGAAAAAGTTCGAGCTGCCGACGATGCCGGCCTCGATGCGCAAGTTCGGCGGCCAGATGGGCGGCGCCTTGCCGGCCAGCGTCAAGAAGCAGATCGCCAAGGCGATCGGCGCACCGCTGTCGGTGCTCGGCTACATCACCAACCGTGCCGTGTTCGACGACAAGAACGCCCGCGCCGCGCTCAAGGGCACCGGCATCAAGTGCCCGGAATTCCGCGAGTACGCGAAGCACCTGTGGTCGTACTGGGAGCAGTTCCTCGACATCCACTACGAGCCGAGTGCCGAGCTGATCGCCAAGGTGAAGGGCAAGGTGATCCTGGTCACCGGCGCCTCATCGGGCATCGGCTTCACCACCGCGAAGAACCTGGCGATTGCCGGCGCCCGGGTCATCCTGGTGGCGCGTACCGAATCGAACCTGATCGAAACTCAGGAGATCATTTCCCGGGCCGGCGGCGAGAGCTATGTCTACCCCTGCGATCTGATCGACATGAAGGCGATCGACGCCATGGCCGCGAAAGTGCTGCGCGACTTCGGCCACGTCGACATCCTGATCAACAACGCCGGCCGCTCGATCCGCCGCGCAGTGATGGAAAGCTTCGACCGCTTCCATGACTTCGAGCGGACCATGGAGCTGAACTACTACGGCGCCGTGCGCCTGATCATGGCGCTGCTGCCGACGATGACCGCGCGCAAGAACGGCCACATCATCAACATCAGCTCGATCGGCGTGCTGGCCAACGCGGCACGCTTCTCGGCCTATGTCGCCTCCAAGGCCGCGCTCGATGCCTTCACCCGCTGCCTGTCGGCCGAAGTGAAAGGCAGCAACATCCGCACCACGGTGATCTACATGCCGCTGGTGCGCACGCCGATGATCGCTCCGACCAAGATCTACAGCTACGTGCCGACCTGGTCCCCGGACGACGCCGCCGACACCGTGATCAAGGCGATCCTCGACGAACCGAAATCGATCGCGACAACTCTGGGTACTGCCGCTGCGGTCAGCTATGCGATCTGGCCGAAGGTCAACGACTACATCCTGTCGAAAGGCTTCCAGCTGTTCCCGTCATCAACCGCCGCACGTGGCTCGAAGGACAAGGACTCGAAGGCCGACAAGCCGACCCTCGAACAGGTGGTGTTCGCGAATGTGTTCAAGGGCGAGCATTGGTAA
